# 116 Relationship between Smartphone-tracked Physical Activities and Psychosocial Outcomes for Burn Survivors Living in the Community

**DOI:** 10.1093/jbcr/irad045.089

**Published:** 2023-05-15

**Authors:** Huan Deng, Cailin A Abouzeid, Lauren J Shepler, Mary D Slavin, Juan P Herrera-Escobar, Lewis Kazis, Colleen Ryan, Jeffrey Schneider

**Affiliations:** Spaulding Rehabilitation Hospital, Harvard Medical School, Boston, Massachusetts; Spaulding Rehabilitation Hospital, Harvard Medical School, Boston, Massachusetts; Spaulding Rehabilitation Hospital, Harvard Medical School, Boston, Massachusetts; Department of Health Law, Policy and Management, Boston University School of Public Health, Boston, Massachusetts; Brigham and Women’s Hospital, Harvard Medical School, Boston, Massachusetts; Boston University School of Public Health, Rehabilitation Outcomes Center at Spaulding, Boston, Massachusetts; Shriners Children's-Boston, Massachusetts General Hospital, Harvard Medical School, Boston, Massachusetts; Spaulding Rehabilitation Hospital/Harvard Medical School/Rehabilitation Outcomes Center at Spaulding, Boston, Massachusetts; Spaulding Rehabilitation Hospital, Harvard Medical School, Boston, Massachusetts; Spaulding Rehabilitation Hospital, Harvard Medical School, Boston, Massachusetts; Spaulding Rehabilitation Hospital, Harvard Medical School, Boston, Massachusetts; Department of Health Law, Policy and Management, Boston University School of Public Health, Boston, Massachusetts; Brigham and Women’s Hospital, Harvard Medical School, Boston, Massachusetts; Boston University School of Public Health, Rehabilitation Outcomes Center at Spaulding, Boston, Massachusetts; Shriners Children's-Boston, Massachusetts General Hospital, Harvard Medical School, Boston, Massachusetts; Spaulding Rehabilitation Hospital/Harvard Medical School/Rehabilitation Outcomes Center at Spaulding, Boston, Massachusetts; Spaulding Rehabilitation Hospital, Harvard Medical School, Boston, Massachusetts; Spaulding Rehabilitation Hospital, Harvard Medical School, Boston, Massachusetts; Spaulding Rehabilitation Hospital, Harvard Medical School, Boston, Massachusetts; Department of Health Law, Policy and Management, Boston University School of Public Health, Boston, Massachusetts; Brigham and Women’s Hospital, Harvard Medical School, Boston, Massachusetts; Boston University School of Public Health, Rehabilitation Outcomes Center at Spaulding, Boston, Massachusetts; Shriners Children's-Boston, Massachusetts General Hospital, Harvard Medical School, Boston, Massachusetts; Spaulding Rehabilitation Hospital/Harvard Medical School/Rehabilitation Outcomes Center at Spaulding, Boston, Massachusetts; Spaulding Rehabilitation Hospital, Harvard Medical School, Boston, Massachusetts; Spaulding Rehabilitation Hospital, Harvard Medical School, Boston, Massachusetts; Spaulding Rehabilitation Hospital, Harvard Medical School, Boston, Massachusetts; Department of Health Law, Policy and Management, Boston University School of Public Health, Boston, Massachusetts; Brigham and Women’s Hospital, Harvard Medical School, Boston, Massachusetts; Boston University School of Public Health, Rehabilitation Outcomes Center at Spaulding, Boston, Massachusetts; Shriners Children's-Boston, Massachusetts General Hospital, Harvard Medical School, Boston, Massachusetts; Spaulding Rehabilitation Hospital/Harvard Medical School/Rehabilitation Outcomes Center at Spaulding, Boston, Massachusetts; Spaulding Rehabilitation Hospital, Harvard Medical School, Boston, Massachusetts; Spaulding Rehabilitation Hospital, Harvard Medical School, Boston, Massachusetts; Spaulding Rehabilitation Hospital, Harvard Medical School, Boston, Massachusetts; Department of Health Law, Policy and Management, Boston University School of Public Health, Boston, Massachusetts; Brigham and Women’s Hospital, Harvard Medical School, Boston, Massachusetts; Boston University School of Public Health, Rehabilitation Outcomes Center at Spaulding, Boston, Massachusetts; Shriners Children's-Boston, Massachusetts General Hospital, Harvard Medical School, Boston, Massachusetts; Spaulding Rehabilitation Hospital/Harvard Medical School/Rehabilitation Outcomes Center at Spaulding, Boston, Massachusetts; Spaulding Rehabilitation Hospital, Harvard Medical School, Boston, Massachusetts; Spaulding Rehabilitation Hospital, Harvard Medical School, Boston, Massachusetts; Spaulding Rehabilitation Hospital, Harvard Medical School, Boston, Massachusetts; Department of Health Law, Policy and Management, Boston University School of Public Health, Boston, Massachusetts; Brigham and Women’s Hospital, Harvard Medical School, Boston, Massachusetts; Boston University School of Public Health, Rehabilitation Outcomes Center at Spaulding, Boston, Massachusetts; Shriners Children's-Boston, Massachusetts General Hospital, Harvard Medical School, Boston, Massachusetts; Spaulding Rehabilitation Hospital/Harvard Medical School/Rehabilitation Outcomes Center at Spaulding, Boston, Massachusetts; Spaulding Rehabilitation Hospital, Harvard Medical School, Boston, Massachusetts; Spaulding Rehabilitation Hospital, Harvard Medical School, Boston, Massachusetts; Spaulding Rehabilitation Hospital, Harvard Medical School, Boston, Massachusetts; Department of Health Law, Policy and Management, Boston University School of Public Health, Boston, Massachusetts; Brigham and Women’s Hospital, Harvard Medical School, Boston, Massachusetts; Boston University School of Public Health, Rehabilitation Outcomes Center at Spaulding, Boston, Massachusetts; Shriners Children's-Boston, Massachusetts General Hospital, Harvard Medical School, Boston, Massachusetts; Spaulding Rehabilitation Hospital/Harvard Medical School/Rehabilitation Outcomes Center at Spaulding, Boston, Massachusetts; Spaulding Rehabilitation Hospital, Harvard Medical School, Boston, Massachusetts; Spaulding Rehabilitation Hospital, Harvard Medical School, Boston, Massachusetts; Spaulding Rehabilitation Hospital, Harvard Medical School, Boston, Massachusetts; Department of Health Law, Policy and Management, Boston University School of Public Health, Boston, Massachusetts; Brigham and Women’s Hospital, Harvard Medical School, Boston, Massachusetts; Boston University School of Public Health, Rehabilitation Outcomes Center at Spaulding, Boston, Massachusetts; Shriners Children's-Boston, Massachusetts General Hospital, Harvard Medical School, Boston, Massachusetts; Spaulding Rehabilitation Hospital/Harvard Medical School/Rehabilitation Outcomes Center at Spaulding, Boston, Massachusetts

## Abstract

**Introduction:**

Following burn survivors in the community is critical to understand their problems and needs. Physical activities automatically recorded by smartphones have shown to be a convenient and burdenless method for post-discharge assessment. However, it is unclear if physical activities are associated with burn survivors’ physical and psychosocial outcomes. This study aims to examine the relationship between smartphone-tracked physical activities and scale-based outcomes.

**Methods:**

Adult burn survivors living in the community were included in this study. A mobile application was downloaded to participants’ smartphones and automatically recorded daily number of steps, travel miles and activity minutes. Participants received weekly surveys assessing pain (Patient-Reported Outcomes Measurement Information System (PROMIS) Pain Intensity and Interference), depression (Patient Health Questionnaire (PHQ-8)), anxiety (PROMIS Anxiety) and social participation (Life Impact Burn Recovery Evaluation (LIBRE) Social Interactions and Social Activities). Correlation coefficients between physical activities and survey results were examined and interpreted as “very weak” (|r|≤0.19), “weak” (0.20≤|r|≤0.39), “moderate” (0.40≤|r|≤0.59), “strong” (0.60≤|r|≤0.79), and “very strong” (0.80≤|r|≤1.00).

**Results:**

There were 24 burn survivors with an average of 24 years after injury included in this study. The 180-week data collected between November 2021 and August 2022 was analyzed. Daily number of steps, travel miles and activity minutes separately showed relationships with PROMIS Pain Intensity (r=-0.312, p< 0.001; r=-0.247, p=0.001; r=-0.239, p=0.001), PROMIS Pain Interference (r=-0.375, p< 0.001; r=-0.318, p< 0.001; r=-0.291, p< 0.001), PHQ-8 (r=-0.403, p< 0.001; r=-0.357, p< 0.001; r=-0.311, p< 0.001), PROMIS Anxiety (r=-0.310, p< 0.001; r=-0.209, p=0.007; r=-0.150, p=0.054), LIBRE Social Interactions (r=0.272, p< 0.001; r=0.291, p< 0.001; r=0.328, p< 0.001) and LIBRE Social Activities (r=0.381, p< 0.001; r=0.414, p< 0.001; r=0.404, p< 0.001).

**Conclusions:**

Daily number of steps demonstrated a moderate correlation with depression while both daily travel miles and activity minutes showed moderate correlations with social participation. The results support that smartphone-tracked physical activities are related to burn survivors’ psychosocial outcomes.

**Applicability of Research to Practice:**

This study may inform future use of smartphone-tracked physical activities to understand and track burn recovery and ultimately improve care.

